# Social inequalities, sexual tourism and HIV in Cartagena, Colombia: an ethnographic study

**DOI:** 10.1186/s12889-020-09179-2

**Published:** 2020-08-08

**Authors:** M. C. Quevedo-Gómez, A. Krumeich, C. E. Abadía-Barrero, H. W. van den Borne

**Affiliations:** 1 Universidad del Rosario, School of Medicine and Health Sciences, Bogotá, Colombia; 2 Maastricht University, Department of Health, Ethics and Society, Maastricht, The Netherlands; 3 University of Connecticut, Anthropology & Human Rights Institute, Storrs, CT USA; 4 Maastricht University, Department of Health Promotion, Maastricht, The Netherlands

**Keywords:** Racialised sex tourism, HIV, Social inequalities, Cartagena, Colonial past

## Abstract

**Background:**

Cartagena, Colombia’s main port on the Caribbean Coast, reported an HIV incidence of 7.5 per 100,000 inhabitants in 2007 with 90.0% transmission by heterosexual contact and 70 identified as women with a stable partner. Studies across Colombia illustrate that HIV infection relates to social inequalities; most people with HIV live in poverty and have minimal access to health care, education, and secure jobs. The purpose of this article is to analyse the relationship between social inequalities, sexual tourism and HIV infection in Cartagena, Colombia.

**Methods:**

Data come from a five-year participatory ethnography of HIV in Cartagena in the period 2004–2009, in which 96 citizens (30 of whom were living with HIV) participated in different data collection phases. Techniques included participant observation, in-depth interviews and thematic life histories. Out of this material, we selected three life histories of two women and a man living with HIV that are representative of the ways in which participants expressed how social inequalities make it virtually impossible to engage in safe sex practices.

**Results:**

At stake is the exchange of condomless sex for goods within the widespread sexual tourism networks that promote an idealisation of dark-skinned men and women as better sexual performers. Our results illustrate the complex interplay of social inequalities based on class, skin colour, gender and sexual orientation. Furthermore, they suggest a synergistic effect between poverty, racialization, and gender inequalities in the historical maintenance of social dynamics for a fruitful growth of a sexual tourism industry that in turn increases vulnerability to HIV infection.

**Conclusions:**

Although the convergence of social inequalities has been thoroughly reported in the literature on social studies of HIV vulnerability; distinctive dynamics are occurring in Cartagena, including a clear link between the contemporary globalised sexual tourism industries and a racialised social structure - both having historical roots in the colonial past-.

## Background

Engaging in sexual practices with “locals” while traveling has been part of human history [1, 2]. As noted by McKercher & Bauer ([3]:3) “tourism, romance, love, and sexual relations have been inextricably linked since the earliest days of travel.” For as long as people have been traveling, they have been engaging in romantic and sexual encounters of various types. Sometimes sex or the prospect of sexual encounters at the destination or along the way plays a central role in the decision to travel. Sexual tourism is the term used to capture “individuals who plan their travel for the purposes of obtaining sex” ([4]:122). Sexual tourism exists around the world and different studies indicated an association between sexual tourism and a sexualized racialization of populations related to specific colonial histories [1, 5, 6]. Indeed, the literature reports on different characteristics attributed to sexualised bodies in Africa [7–9], Asia [10–13], and Latin America [14–22], depending on specific sexual ideations that tourist from Europe and North America have. Besides, Europe [6, 23, 24] and North America also report [1, 6, 25, 26] cases of sexual tourism. Besides the exploitation of bodies from the global south, the sexual tourism industry exposes local populations to specific diseases or potentiates the relationship between social inequalities, violence and health as seen in the sexual abuse of adults and children [11, 15, 22, 27] and their heightened vulnerability to sexually transmitted infections [12, 16, 28].

Studies on HIV risk and tourism in Latin America, Thailand and South Africa have illustrated that individuals employed in the tourism industry, both male and female sex workers, are highly vulnerable to HIV infection due to the intersection between social inequality, poverty, and the racialization of people’s bodies [1, 17, 21, 22, 29, 30]. Importantly, in these unequal relationships, tourists might demand sex without a condom [12, 21, 31, 32]. Hobbs et al. [10] linked gender inequalities to hegemonic masculinity in setting up female sex workers´ HIV vulnerability in Thailand. Bauer’s description of *bricheras/os* (romantic and sexual relationships with tourists as a means of income) underscores how HIV vulnerability results from links between social inequalities and local tourism in Cuzco, Peru [18]. Studies in the Caribbean region suggest that the insidious junction of racism -product of the colonial history-, poverty and gender inequality, as described by Farmer [33] among others, is reinforced by the powerful economic force of the sexual tourism industry. Padilla [28, 34] further argues that global inequalities create a market for sexual encounters between sexual tourists and Dominican men, who use their bodies to create temporary sexual identities that serve the demand of the tourist. He explains that these expressions of agency may help overcome marginalisation but contribute to riskier sexual behaviour. Cabezas [22] argues that Dominican women who are part of the sexual tourism industry suffer from gender, economic, sexual and racial inequalities that impede them from negotiating condom use effectively in comparison to Cuban women, who are more likely to negotiate sexual safety given Cuba’s larger social capital.

Colombia stands out as one of the countries with higher rates of sexual tourism and child abuse around the world [6, 23, 24]. Situation that could possibly have increased in the past 2 years after the signature of the peace agreement and the current president promotion of Colombia as an international tourist hub [35]. Supported by the existing literature [14, 22, 28], we ask for the intersection between gender, race, social inequality and HIV vulnerability in Colombia. Studies across Colombia do suggest that HIV infection is related to social inequity and political economic forces since most people living with HIV are poor and have minimal access to health care, education, and secure jobs [36–39]. Our study took place in Cartagena -the country’s main port city on the Colombian Caribbean Coast-, which reported an HIV-incidence of 21 per 100,000 inhabitants in 2009, much higher than the national average, of which 95% were acquired by heterosexual contact and 70% were identified as women with a stable partner [40]. Not only does Cartagena have a high level of social inequity [41], with 49% of its population living under the poverty line, 60% of whom are Afro-Colombian women [42], it has also been an important tourist destination to the point that it was declared an international tourist district in 2004. Hence, we ask about the interplay between large social inequities, the growing sexual tourism industry that reports child abuse in Cartagena [15] and heterosexual transmission of HIV among men and women [43] and how this interplay adds to HIV vulnerability among Cartagena’s citizens.

Cartagena’s social reality regarding the intersection between gender, race, social inequality and HIV vulnerability is not extensively studied. Therefore, with our study we intended to explore how Cartagena’s citizens perceive and experience social inequalities and the sexual tourism industry in relation to the HIV epidemic.

## Methods

**Fig. 1:**
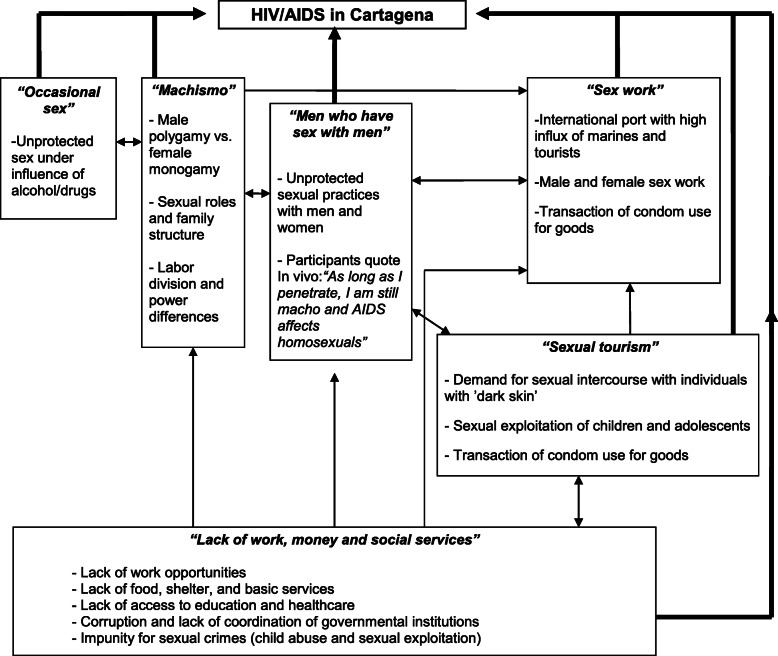
Local-Scientific diagram of elements related to HIV infection in Cartagena. Adapted from: *Quevedo-Gómez, María Cristina, Krumeich, Anja,Abadía-Barrero, César Ernesto,Pastrana-Salcedo, Eduardo Manuel, van den Borne, Hubertus (2011) Structural actions toward HIV/AIDS prevention in Cartagena, Colombia: a qualitative study. Rev Panam Salud Publica;30* (1) *65–73,july 2011. Retrieved from*http://www.scielosp.org/scielo.php?script=sci_arttext&pid=S1020-49892011000700010

The data presented here are part of a five-year participatory ethnography of HIV in collaboration with 96 citizens of Cartagena. Different methods and data collection techniques were used, including 40 in-depth interviews (average duration: 1 h) and 30 thematic life histories of inhabitants living with HIV (average duration: 1.5 h) on the scenario of HIV vulnerability were collected and summarised in a preliminary diagram. This preliminary diagram was evaluated and complemented through group discussions with a total of 36 individuals: i.e., key representatives of local governmental and nongovernmental organizations, and other inhabitants who were interested in the epidemic or affected by it. The evaluation led to a ‘Local-Scientific diagram’ (see Fig. 1: Local-Scientific diagram of elements related to HIV infection in Cartagena) of social determination of HIV infection [44] that was further discussed by key stakeholders in Cartagena, who identified sexual tourism as one of the most important determinants of the city’s epidemic.

For this paper, we selected three life histories exemplary of the interplay between social inequalities, sex without a condom in exchange for goods, sexual tourism and ultimately HIV infection depicted by all participants in the ‘Local-Scientific diagram’. The life histories give an in-depth insight into how different aspects particular to daily life in Cartagena intertwine shaping inhabitants’ vulnerability to infection. The 15 men and 15 women living with HIV who shared their life histories during an initial 7-month fieldwork period in 2006–2007 were selected by the first author and by the key initial participants through purposeful sampling [45]. In the life histories, people were invited to describe their life trajectory, to report on how and when they were diagnosed, how they thought they were infected, what they knew about HIV before their diagnosis, and what they had experienced after the diagnosis. Our analytical interpretations relied on concepts from medical Anthropology studies on HIV (e.g., power, gender, ethnic and other differences related to HIV infection), sociology (e.g., social construction of sexual meanings and practices), and political economy (e.g., reinforcement of structural inequalities through globalised political and economic processes).This research project was submitted to and approved by *Centro de Estudios Socioculturales e Internacionales* ethics committee at *Los Andes University* Accordingly, all research procedures followed ethical norms as laid out in resolution # 8430–1993 by the Colombian Ministry of Health and all participants accepted and signed written informed consents. The open interview questions were developed for this study by the first author (English version of the questions are uploaded as a supplementary file). The material from the three life histories, as well as the rest of the collected material presented in this article has been translated and edited by the first author, and remain in a secure location under the access of only the main researcher. The datasets used and/or analysed during the current study are available from the corresponding author on reasonable request.

## Results

### We lived off her money: family dynamics within sexual tourism

M. P.^1^ is a 31-year-old woman living with HIV. She is a home-staying mother with 4 years of primary school education. She lives in extreme poverty and self-identifies as “black.” M.P. told us:

*“I was born here in the slums of Cartagena. I lived with my mother and my five brothers and sisters. We went through many days of hunger. My father never gave money to the family; my mother took care of our food and education, trying to survive day by day. When my mother fell ill, I had to leave school and start working to help her. At that time, I met the father of my first kid. After that man got killed, I met my current husband. I have been living with him for 12 years in one of the slums next to the hill. We have three kids. We have electricity but since my house is up-hill we do not have access to water. We still do not have sewage; you know that poor neighbourhoods, where black people live, are always forgotten.”*

Participants, that share the perspective of this woman about the poor living conditions of the “black” population, indicate that in Cartagena the colour of the skin is associated with the social position of inhabitants. This, in turn, implies that “non-white” inhabitants grew-up in extreme poverty with limited access to education, health, work and other social opportunities and continue to live in these marginalized conditions. This perspective reported also by Streicker [46], who describes how class and ‘race’ in Cartagena function as interlocking categories to maintain the status quo of the authority of “non-blacks”. Streicker [47] illustrates how the social position in this city closely links to skin colour, with “whites” occupying the highest position, and “blacks” in the lowest position.

The above-described racialised structure [48] of Colombian society, reported also by Castro-Gómez [49], who attributed it to the pervasiveness of a colonial racial order. Spaniard conquerors initiated a clear differentiation between social groups based on ethnic background and religious beliefs. While, Native-Indians were considered worthy of Christian conversion through crusades, and could be granted the status of servants, debates about whether African populations were human, set up the political and religious context of the establishment of black slavery [49]. Thus, racial mixing forced a reconfiguration of social classes and created a coloured hierarchy from “white” to “brown” (mestizo and mulato) to “black”, in which the proof of white descent marked contrasts in possessions, prestige and power [49]. Cartagena’s colonial past is particularly interesting given that as the main port city, the arrival, trade and settlement of African populations was very significant. Differentiations in access to goods and social opportunities based on ethnic background are still evident in the current distribution of inequality and poverty in the city. According to Pérez and Mejía [42], to date, 80% of Cartagena’s inhabitants who self-identify as “black”, *“mulato”* or *“mestizo”* have restricted access to work opportunities and live in the poorest areas of the city, in contrast to the minority of inhabitants who self-identify as “white”. The latter data suggest a permanence of colonial power relations that resulted in a current racialised social structure in the city, in which the ethnic background still relates to oppression and discrimination, despite dynamic modifications over time.

The following section from M. P.´s story shows how historical marginalisation underlying contemporary racialised social inequality has implications for HIV infection among men and women in Cartagena.

*“It has been six months since I got to know that we are sick; we got sick through him, my husband. He got very sick and was very slim. He brought food and money home but he got sick. I have to tell you that only this year has been a happy year for me because he is at home much more. He has been always a womaniser. Five years ago he worked as a bar tender in a hotel and as I told you he lived his life as if he was alone, although he lived with me! In that hotel he met a 52 year old woman from Norway who came to find a black man to have sex with. Oh, yeah! He is 28 years old. She had a lot of money and bought him a motorcycle. He started working as a motorbike taxi-driver along with his job at the hotel. She gave him a lot of things! Then he started to get sick. I did not know that he was with her, until a friend of mine told me that she saw my husband with a foreign woman. So, I asked my husband if that was true and he said: “Yes, I am together with that woman but I want you to help me and support the situation because this is a good way to get what we need for our family.” I understood and accepted. He had a double life; he lived with me and with her; in his way: 3 days with me and two with her. This went on for 4 years. During that time we have been financially fine because she gave him all he wanted. She was buying the food for all of us, paying the school fees for the kids; she practically bought everything we needed. We lived off her money. She knew about me and the kids but she wanted to take him to Norway. He did not accept. […] The doctor said: “I have the impression that you are HIV positive.” That was the first shock for both of us! So, we did the test and yes, we both are positive. We still do not like to use a condom, it is difficult. I trusted him, he is my husband. I never thought AIDS could happen to us. Well, he was a womaniser but he always came home and was sweet to me, then we stayed together without protection. I trusted him, he is my husband. Now he is not with that woman, I do not know if she was the one who infected him, he does not know. Now he cannot work.”*

This section of M.P.’s story illustrates how social inequality and poverty –products of a racialised social structure- leave almost no alternatives for survival, aside from the acceptance of sexual tourism as the main source of income for this family. On the one hand, the marginalisation of these “black” families creates vulnerability by reducing their access to goods and services, thus increasing the appeal of the alternative sources of income provided by sexual tourism. On the other hand, the sexual preference for individuals with “black skin” among tourists [21, 22, 34] has created an informal economy in the city around the exchange of sex for goods and money that is evident in this particular life history, in which M.P. accepts that her partner exchanges sexual relationships for basic goods with a tourist.

The story of M.P. represents the cases of other local women equally dependent on their partners. It shows that the vulnerability to HIV in Cartagena is related to economic, racial and gender differences. Despite M.P.’s compliance with female traditional gender roles within machismo; in which women protect themselves either through their own fidelity or through the reliance on their partner’s protective role (i.e., the man will use condoms with other women to protect his main partner), she still acquired HIV. The traditional male sexual role within machismo encourages M.P.’s husband to both multiple sexual partners and being a responsible provider of the household’s economic needs. Thus, the machismo norms and economic dependency expressed in M.P.´s reliance on her partner and her partner’s duty to provide goods situate the family in a vulnerable situation with respect to HIV infection [50].

This first life history illustrates how HIV infection occurs in the insidious interplay between poverty, limited job opportunities for “black people”, the male and female sexual roles within machismo, and a sexual tourism industry that promotes sexualised stereotypes linked to skin colour. What is striking about this history is how the attempts of this particular family to overcome marginalisation and poverty led this couple to the acceptance of sexual tourism as a unique alternative for economic survival. Although poverty is known to go hand in hand with increased vulnerability to HIV infection, in this case, attempts to escape poverty also increased vulnerability to HIV infection.

### This works like a market, a business: sexual orientation, machismo and sexual tourism

N.A. is a 36-year-old man living with HIV. He is an unemployed professional who self-identifies as “gay” and “brown.” N.A. shared his story:

*I always felt attracted to men, never had sex with a woman. I never knew why I liked men. I do not have feminine manners, I dress like a man but you know that in this society, the one who has no girlfriend at the age of 17 is a ‘marica’,*^2^*thus people started calling me ‘marica’. I wish I could be a man who likes to be with women but I have to accept myself; only my family and close friends know. In this ‘machista’ society you have to keep that silent people talk a lot here. It is important to protect your job; especially now that I lost mine. [Silence] As you must know by now, blacks, gays and people living with AIDS are not wanted for good jobs.*

In this section, N.A. presents his reduced job opportunities and general social disadvantage as a result of the combination of being “non-white”, “gay” and living with HIV. Given his experience with homophobia, he opts for hiding his sexual orientation. The stigma management strategy of keeping silent about his sexual orientation for social and economic protection was described also in Mexico [51] and the Dominican Republic [34]. The experience with constrained job opportunities shows how, in this case, social inequalities based on skin colour merge with discrimination based on sexual orientation, due to homophobia (resulting from machismo traditions) and with discrimination based on sero-status. This combination of social processes enhances vulnerability through unemployment and poverty. Furthermore, as illustrated in the previous story, these social processes increase the need for alternative income sources, such as sexual tourism. As the next section will show, vulnerability is also related to dependencies on “gay” and heterosexual sexual networks, which increase the likelihood of engaging in sex without a condom.

*“How do I get to know men? We go with my friends to the gay bars; there you can always find men. Here in Cartagena, it is becoming like a trend that black and brown young men, and I mean machos that like women, go to the gay bars to make their business. They go there just to get all the alcohol, food, dance, sex and money that they want. They sit there; seduce you and when they see that you are into sex, they tell you: “You know that this costs money, right?” Then, you accept because you like the man. They are young; they must be 17 or 18 years old. You see that a lot here, young guys with gays! I guess they just do it for the money or for having someone invite them to eat a meal and drink rum. Many say that they need the money to help their sick mother or they tell you family dramas like: “My father was killed and I have to help mum” but … who knows, maybe they just want the money to invite their girlfriend for a nice night out. Although, sometimes you notice that they are really going hungry. This works like a market, a business. They are becoming very experienced, they know where to find us [“gay” men]; they offer you, like, an all-inclusive package for a moderate price. They ask for more money if you want to make it without a condom and the only condition is that they penetrate you. They say: “My business is to penetrate ‘maricas’” My gay friends and I almost always end up paying for sex. The minimum price is $col 25.000 [US$ 13.30]. When I had a good job, I could pay these guys but now… [silence] Other men who you find in those places are machos who are already married and have kids but once in bed you get surprised they are more ‘maricón’ than you! These men hardly ask for a condom, they just enjoy, they want everything.”*

This narrative reports on how homophobia hinders men’s possibility to openly express sexual preferences. By doing so, it creates dependence on homo-social networks to fulfil emotional and sexual needs. It also illustrates how the racialised marginalisation leads to extreme poverty and the need for alternative sources of income. N.A. tells us of an informal sexual market around the gay sexual networks, where self-identified heterosexual young men exchange sex with older “gay” men for goods and money. Young boys profit from the sexual and emotional needs of “gay” men by crafting temporary sexual identities/practices as ‘machos that penetrate *maricas* for money’. Therefore, these young men also become vulnerable because they construct their sexuality under economic pressure. Under these circumstances, both self-identified young heterosexual men and “gay” men find themselves in scenarios of prostitution and HIV vulnerability through the practice of condomless sex (see section below). Similar constructions of temporary sexual identities among men who have sex with men and women (MSMW) to serve the sexual tourism demand has been described in the Dominican Republic [34]. As the next section shows, homophobia, a lack of job opportunities and poverty lead not only to this local sexual market, but also stimulate the exchange of sex for goods in the context of national and international sexual tourism. Sexual tourism, in turn, augments vulnerability to HIV infection.

*“Another way of getting men is in the tourist season. Then, we all get our good share, not only the young guys. I have to admit that I enjoy being invited by tourists. They offer you good food, nice drinks; they bring you to expensive hotels and make you feel good. You know that as a brown and unemployed man, I would have not had the chance to enjoy this luxury. Well, there is also another type of tourist, mainly the internationals; they are white men who come for drugs and sex. They like brown and black men and women; they say that we know how to have sex best. I know some women who charge them a lot of money for the night and I also know that the price without a condom is very high! You can live almost one month off of that money! Many people live the rest of the year with the money they make in the tourist season. That I do not! [sic] I only enjoy when they invite me and have fun with them. […] I must have gotten my disease on one of those days that I did not use a condom, but who knows who it was? I wonder if these young boys get any information or even think about it. Once, one of these young boys told me that he did not need a condom because he was the macho [the person who penetrates]. I also worry for the machos with a wife because they sleep with men and women without any protection.”*

Other participants, who self-identify as “brown” and, who exchanged sex for goods and money, shared this participant’s perspective of the “advantages” of the tourist season. This perspective refers to the relevant role of the stereotype of “brown and black” inhabitants as better sexual performers. This opinion also illustrates how the exchange of sex with tourists provides temporary access to specific goods, such as expensive/exclusive restaurants, bars and hotels, resulting in a temporary social upgrade, which “brown and black” inhabitants would otherwise hardly experience.

This life history illustrates the interplay between the racialization and heterosexualisation^3^ of social and work opportunities, and a sexual tourism industry centred on sexualised stereotypes linked to skin colour in the construction of vulnerability to HIV infection for both “gay” men and young men self-identified as ‘machos that have sex with men for money’. For both types of men, the lack of access to goods and a long-term social exclusion open the doors to exchange of sex for money or goods, as either a survival strategy or a strategy for social upgrade.

### I see how bad my situation is and I cry: a housewife in prostitution

E.M. is a 30-year-old woman living with HIV. She is a woman, who had no opportunities of formal education, and lives in extreme poverty and self-identifies as “black.” E.M. shared her life history:

*“I was born here in this poor agglomeration of houses on the beach. Here, I met the father of my two-year-old boy. He made me a rancho [wood house] in the slums. I lived there for 8 years but he treated me very badly! He used to beat me with a stick. Once, I even lost a tooth. He used to get home drunk and started insulting me and kicked and kicked [silence and tears]. One day the words of my neighbours gave me the strength to leave him. They used to tell me: ‘Are you going to let him kill you just to keep this rancho?’ I came back to my mother’s house but she is not helping me with the kid. I have to find the money by myself. The father is not giving me money for the kid. I am a housewife, so that is what I can do. I can help with the household, or clean in a restaurant, anything! I have asked all my neighbours and friends to help me find a job to support myself and my kid. My boy starts school on Monday but I have nothing to pay his fees. I wish I could find something [she cries].That is why I am here doing this [silence]. If people want me to iron or do the laundry or to work on the beach doing massages, I am happy to do that but sometimes men come to you for sex with tourists and then, I cry, I see how bad my situation is and I cry, what else can I do? I am not in prostitution but if they give me $col. 20.000 [US$ 11] I take the money and have sex with the man. I feel obligated by my situation. I have days in which I cannot make any money, not even for a meal. The fishermen that come in the morning to the beach see that I am hungry, so they offer to give me a fish if I sleep with them. Then, I make the effort because I can feed my kid with that fish. […] On this beach, there is a man who calls us [women] when there is work. He rents the room for $col. 5.000 [US$ 2.75]. The men pay him and in addition they pay us for the sex. The men that come to this beach for sex are construction workers who come from the nearby rich neighbourhoods, and also tourists -mostly cachacos [meaning everyone who comes from the interior of the country] but also men who come from very far, for example Italy, Spain, and Canada-. These foreigners like to have sex with us, the black girls. The foreigners say that they have chosen us from a catalogue on the internet. I have heard that they buy, like, a tourist package that includes the woman. I do not know how that works.”*

As in the first life history, E.M.’s story also shows how poverty, which is historically related to marginalisation of the “black” population, underlies unemployment and lack of access to goods and services, including education. Similar to the life histories of the other women, marginalisation also merges with gender inequalities and leads to increased vulnerability, confirming previous studies on machismo and HIV in Colombia [39, 50]. E.M’s story provides an example of how, due to machismo social norms, women who leave their partners are rejected by their families because of a failure in their role as women. It also shows how women, once confronted with the need to be economically independent, face a lack of job opportunities due to racial inequality and economic hardship. These women end up exchanging sex for food, money or goods with locals, national and international tourists. As illustrated in the next section of this life history, the picture becomes more complex when gender inequalities and marginalisation enter the picture, merging with a sexual tourism industry that market “Colombian black women”.^4^

*“[…]About AIDS, I do not know much, I have heard that is a bad disease; it makes you vomit and have diarrhoea until you get very slim and die. Once I heard the health worker talking about it in the slums where I lived. They came to talk about it and said that you had to use a condom when you made love. Since I had my husband at that time, I did not worry because we do not use a condom with our man. Having sex with your husband is safe… This man [the procurer of prostitutes] sells condoms cheaper than in the pharmacy (i.e., $col. 1.000 [US$ 0.55].) so, I can buy condoms but some men [the clients] do not want to use a condom. When men tell me: ‘I am not doing it with a condom’, then I ask again but sometimes if I have not made any money for the day then, I have to accept it without a condom. Only once I rejected someone, because the man had a “thing” that smelled terribly, he must have been rotten. […] Until now I still do not have medication because I do not have insurance, well I do not have a citizen card yet, I only know I am positive because I had to pay for the test.”*

E.M.’s story clearly illustrates the exploitation of women by sexual commerce on both local and national levels (e.g., low scale prostitution) as well as on a global level (e.g., the sexual tourism industry), and its consequent detriment to the health of this participant. This is in line with Hobbs’s [10] study on transactional sex in Thailand, which links gender inequalities resulting from hegemonic masculinity to HIV vulnerability among female sex workers.

The vulnerability to HIV infection in Cartagena shows how gender, sexual and racial inequalities relate to the poor performance of the government, as evident in the lack of access to education, health care and fair work opportunities.^5^ The lack of strong social protection policies in Cartagena, along with minimal job security, and extreme poverty rates of 50% [42], have made the sexual tourism industry an important economic sector. Adding to the previously-discussed racial and gender inequalities, it should not come as a surprise that the international industry of sexual tourism in Cartagena has grown steadily to the point of “diversifying” into child sexual exploitation [15], as in other countries [11, 52].

## Discussion

Our results illustrate how the interplay between diverse social inequalities based on skin colour, gender and sexual orientation, together with racialised sexual tourism, furthers people’s vulnerability to HIV infection in Cartagena.

Although links between racialised sexualities and sex tourism have been clearly established and suggested as possible contributors to HIV vulnerability in Asia, Africa and in Latin America [1, 5, 9, 18, 20, 22, 28, 34], understanding the unique way in which these processes are linked in Cartagena adds to detailed insights in the local HIV epidemic. Cartagena’s colonial history -characterised by pervasive racial inequalities (47)-, along with a globalised sexual tourism industry [1, 5, 6] make it possible to understand a context for HIV vulnerability in which, on the one hand, “white tourists” find “good sex with black people” and, on the other hand, marginalised inhabitants can access some basic goods, money and even experience temporary social mobility.

Not only is the racialised social structure of Cartagena rooted in the colonial past due to the social stratification grounded on racial and ethical origins [17, 30, 41], but the sexual objectification of racialised men and women by the global sexual tourism industry also seems to have roots in colonial history [17]. As illustrated by Edmonds [17] and Bandyopadhyay & Nascimento [20] “black and Native-Indian bodies” were seen by Portuguese colonisers as inferior and exotic, and therefore attractive and erotic. In a postcolonial and psychoanalytic perspective, Fanon [53] describes how “black women and men” in the Antilles and France relate with “white people of the opposite sex” based on their unconscious response to the historical inferiority imposed on them by “white” colonizers, in an attempt to achieve some of the social status historically attached to “white people”. In Cartagena, the feeling of inferiority and the constant striving for social upgrade through whitening was evident in the narratives of women and men who exchanged sex for money and goods. As illustrated by a 25-year-old woman: *“I like to be with foreigners because they are handsome, white, and invite me to expensive places where I could not enter. Sometimes, I do not use a condom because I would not mind to get pregnant by one of them. Then my kids would be brown and not black. He or she would have an easier life!”* Our participant’s daily experience, interpretations and interactions with the different sexual exploitation networks seem to corroborate both the idea of the colonial rootedness of racial ordering [29, 53] and the appeal of the exotic “black” body to international tourists. Consideration of these historical roots contributed to our better understanding of the complexity of the local narratives and its connections with larger historical processes of prevailing inequalities.

The characteristics of our participants (i.e., being raised in poverty, having a “black or brown” identity, no stable income, and women’s economic dependency on sexual partners), suggest that exchanging sex for goods is the result of the complex convergence of social inequalities based on class, gender, skin colour and sexual orientation. A scenario in which the rapidly growing sexual tourism industry augments people’s vulnerability to HIV infection [19, 28, 34]. Depending on the economic needs of a person, the demands of the client, and the cultural constructions around gender and risk (i.e., machismo as a “protective” mechanism for HIV infection) safe or unsafe sex practices take place. Our findings are in line with other studies that report a direct association between HIV infection and gender inequalities product of machismo [37, 54, 55], and illustrate how these inequalities have a clear interaction with poverty and inequalities based on class and ethnic background. Thus, our results also conform to studies that highlight the association between gender, poverty and ‘race’ in the creation of vulnerability to HIV infection [29, 33, 56]. Our findings on sexual exchange for goods and money also illustrate how sexual tourism becomes a survival strategy and a means of social upgrade for racialised men and women, complementing previous studies [1, 9, 18, 20–22, 28].

Furthermore, our results on pervasive heterosexism related to HIV infection support studies in Latin America that show how gender roles imposed by machismo also increase men’s vulnerability to HIV infection in the context of sexual tourism [21, 28]. These results also add the previously mentioned complex interaction between heterosexism, racial discrimination, poverty and sexual objectification of racialised men by the global sexual tourism industry in creating the scenario for HIV infection among heterosexual and “gay” men in Cartagena.

### Limitations of this study

Limitations in the sample: In the first fieldwork, lesbian and transgender groups were not included in the initial sample by key participants. There is a risk that comes with letting the non-scientific participants make decisions about whom to include and not include in the study. The participants might have deliberately left out these groups, as they are not socially accepted. Therefore, in future research it is necessary to target these population groups in order to further explore the diversity of existing sexual practices and identities in the city and investigate a possible relation of these practices to HIV infection. Limitations in the analysis: Due to time constrains, it was not possible to conduct deeper analysis on the role of the performance of the government and its dynamic interplay with the other elements involved in the social determination of HIV infection and vulnerability in Cartagena presented in this dissertation. Therefore, further analysis based on the data collected in this research and additional data is necessary in the future.

## Conclusions

The interplay of inequalities that take place in Cartagena mirrors the global system of inequalities that led to HIV infection discussed previously in the international literature [29, 36, 53, 57–61]. However, the specific insertion of a racialised global sexual tourism industry, within embedded historical structures of racialisation and marginalisation, suggests a historically-driven connection between social inequalities, racialised sexualities and sexual tourism in setting the scene for HIV infection and vulnerability in Cartagena. Consequently, attempts to reduce the spread of the local epidemic and its social impact should address the complex interplay of inequalities and their relation to global sexual tourism that have been exposed in this paper.

## Supplementary information

**Additional file 1.** Interview Guide used and developed for this study.

## Data Availability

The datasets used and/or analysed during the current study are available from the corresponding author on reasonable request at mariacr.quevedo@urosario.edu.co.

## Abbreviations

HIV: Human Inmunodeficiency Virus
AIDS: Acquired Immunodeficiency Syndrome
Nuffic: Netherlands Organization for International Cooperation in Higher Education
Colciencias: Colombian Institute for Science and Technology Development

## Footnotes

1 Given that people living with HIV in Colombia are persecuted by irregular armed forces; in order to protect the identity and security of all participants, initials of pseudonyms instead of full pseudonyms are used.

2 *Marica, maricón*, and *(hombres) gay* (Spanish word) are all derogatory words used in Cartagena that refer to men who have sex with men and men who are suspected of being penetrated by other men.

3 We use the term heterosexualisation here to group the effects of social and work discrimination based on heterosexism within *machismo.*

4 This quote comes from a web advertisement for sexual tourism in Colombia.

5 For an initial discussion on this topic see Quevedo-Gómez MC, Krumeich A, Abadía-Barrero CE, Pastrana-Salcedo EM, van den Borne H. Structural actions toward HIV/AIDS prevention in Cartagena, Colombia: a qualitative study. Pan American Journal of Public Health. 2011; 30 [1]:65–73.
