# Supplementary material for: Social inequalities, sexual tourism and HIV in Cartagena, Colombia: an ethnographic study
Source: BMC Public Health. 2020 Aug 8;20:1208. doi: 10.1186/s12889-020-09179-2 (PMC7414756; doi:10.1186/s12889-020-09179-2)
Supplement: Supplementary file 1 — Additional file 1. Interview Guide used and developed for this study. [file 12889_2020_9179_MOESM1_ESM.pdf]

Interview Guide used and developed for this study

Out of the 70 study participants 30 were people LWH.

With those participants who reported not having taken a test for HIV or had tested negative for HIV, the main researcher conducted an **open-ended interview** (average duration: 1 hour) **that started with one major question: "Please tell me everything you know about AIDS."** Further questions were limited to clarification of particular statements. In some cases the main researcher asked participants to comment on statements brought up by other participants. This approach led to the self-reflection and the critical awareness associated with PAR approaches and resulted in a critical evaluation of each participant's risk of HIV infection in relation to the social context of HIV infection in the city.

Participants LWH were invited to share their **life histories** that were recorded by the main researcher (average duration: 1 hour 30 minutes). **In the life histories, they described their life trajectory, explained how and when they were diagnosed, their interpretation on how they got infected, what they knew about HIV and AIDS before their diagnosis, their opinions on how other people get infected in Cartagena, and what they had experienced after the diagnosis.** Their personal experiences complemented and confirmed data collected through the interviews and gave a comprehensive image of everyday dynamics of HIV infection and vulnerability in the city.

Information extracted from the PhD thesis of the first author: María Cristina Quevedo-Gomez
